# Intermittent Fasting and Metabolic Health

**DOI:** 10.3390/nu14030631

**Published:** 2022-01-31

**Authors:** Izzah Vasim, Chaudry N. Majeed, Mark D. DeBoer

**Affiliations:** 1 Department of Internal Medicine, Section of Hospital Medicine, Wake Forest University School of Medicine, Winston Salem, NC 27101, USA; izvasim@wakehealth.edu; 2 Department of Internal Medicine, Division of Gastroenterology, Wake Forest University School of Medicine, Winston Salem, NC 27101, USA; cmajeed@wakehealth.edu; 3 Department of Pediatrics, Division of Pediatric Endocrinology, University of Virginia School of Medicine, Charlottesville, VA 22908, USA

**Keywords:** intermittent fasting, obesity, metabolic syndrome, type 2 diabetes, ketones

## Abstract

Given the ongoing strain that the obesity epidemic has placed on public health outcomes, new and effective approaches to weight control are needed. One approach to improving weight and metabolic outcomes is intermittent fasting, which consists of multiple different timing schedules for temporary food avoidance, including alternate-day fasting, other similar full-day fasting patterns, and time-restricted feeding (where the day’s food is consumed over a 6-h period, allowing for 18 h of fasting). These feeding schedules have favorable metabolic effects by intermittently inducing the metabolism of fatty acids to ketones. The regimens overall lead to a decrease in weight and have been linked to improvements in dyslipidemia and blood pressure. While more research is needed on longer-term outcomes and this approach should be avoided in particular health conditions, intermittent fasting should be considered as an option for individuals who have a pattern of unhealthy weight gain using standard eating patterns.

## 1. Introduction

Obesity and metabolic syndrome (MetS) are a growing problem all across the globe, and hence there has been an increasing focus among the medical community to come up with innovative therapies to counter their pathophysiological effects [1,2]. Although there has been a remarkable advancement seen in the development of new medical treatments for obesity; recently, there has also been increasing interest towards improving the dietary patterns through various nutritional regimens. Intermittent fasting is one such tool that has been proposed repeatedly by health experts due to its benefits on weight management, cardiovascular health, and oxidative stress [3]. This review article will summarize the consequences of MetS while shedding a light on various types of intermittent fasting, as well as provide a brief summary of the available literature regarding the effects of intermittent fasting on key components of MetS (i.e., insulin resistance, obesity, HTN, and lipids).

## 2. Methods

Prior to conducting the review, a literature search was performed for articles related to the effects of intermittent fasting on metabolism. We used PubMed, MEDLINE, and Google Scholar to search for published articles, including randomized controlled trials, clinical trials, case reports, case series, and review articles between the years of 2010 and 2021. The following keywords were used: “intermittent fasting”, “metabolism”, “metabolic syndrome”, “circadian rhythm “, “diabetes”, “fasting”, “obesity”, “insulin”, “leptin”, “adiponectin”, “insulin resistance”, and “cardiovascular health”. Inclusion criteria consisted of published articles, articles that were available in English, and were full text. Exclusion criteria consisted of duplicates, abstracts, non-English articles, articles did not report outcome measures for any of the previously described variables, and works that were unpublished or unrelated to the topic of interest. Two reviewers independently reviewed abstracts to determine whether the articles met our inclusion criteria. Articles that met the criteria were then further reviewed to determine whether they would be included in our review. After careful review, a total of 66 articles were ultimately chosen and are available in the reference list.

## 3. Metabolic Syndrome and Common Diet Approaches

MetS is characterized as a combination of metabolic abnormalities that includes central obesity, insulin resistance, hypertriglyceridemia, hypercholesterolemia, hypertension, and reduced high-density lipoprotein (HDL)-cholesterol concentrations [4]. It is also associated with other comorbidities including the resultant proinflammatory and prothrombotic states, nonalcoholic fatty liver disease (NAFLD), cholesterol gallstone disease, and reproductive disorders. MetS has been considered to be a chief risk factors in the epidemic of type 2 diabetes and cardiovascular disease in the 21st century [5].

The prevalence of MetS in the United States is estimated at 22–35% [6]. Approximately 35% of adults in the United States have prediabetes, which for 15–30% of them in the absence of intervention will develop type 2 diabetes mellitus (T2DM) within 5 years [7]. The incidence of T2DM worldwide is approaching epidemic levels, correlating with the rising prevalence of obesity. More than 75% of adults in the United States are projected to be overweight or obese by 2030 [8], among whom ~25% will have severe obesity defined by a body mass index (BMI) of ≥35 kg/m^2^ [9]. According to WHO (World Health Organization) data, 17.9 million people die annually from cardiovascular diseases, accounting for approximately one third of all deaths [2,5].

Modifiable and unmodifiable factors contribute to the development of MetS. Modifiable risk factors include smoking, obesity, hypertension, lipid disorders, unhealthy diet, lack of physical activity, and diabetes [10]. The control of these risk factors allows for a reduction of mortality. Lifestyle adjustments such as smoking cessation, increasing physical activity, or maintaining normal body weight, lower the risk of cardiovascular disease [11]. With the growing problem of obesity in the world, diet changes are an important modifiable factor. This has led to an ongoing search for new and effective dietetic solutions aimed at reducing calories and body mass, with the ultimate goal to lessen the effect of MetS. Several studies have evaluated the value of individual dietary patterns has been assessed in several studies [3]. The Western diet includes a high intake of red meat, processed food, refined sugars, and saturated fatty acids. A prospective analysis indicated an 18% higher risk of developing MetS while on a Western diet [12]. The DASH diet (for Dietary Approaches to Stop Hypertension) has proven to reduce BP and CV risk factors; unfortunately, there have been few observational data linking a DASH diet to improvements in MetS [12]. The Nordic diet (named because it is based on foods from Nordic countries) has high amounts of vegetables, fruits, mushrooms, and fish. An RCT of 200 adults with MetS looked at the effects of the Nordic diet (vs. a control diet) for 18–24-weeks. The researchers found that lipid profiles improved significantly while the changes in BMI, insulin sensitivity, and BP were not significantly changed [13]. The vegan diet is a strictly plant-based diet. To date, there have been no large RCT done on vegan diets and MetS, however the protective effects of vegan diets on MetS have been observed in epidemiological studies [5].

## 4. Intermittent Fasting vs. Caloric Restriction

Pursuing an intermittent fasting (IF) diet pattern of eating has been gaining popularity in this realm. Fasting is known to be one of the most ancient traditions in the world, and has been practiced among various communities for either cultural or religious reasons. Interestingly, it has also been used as a healing method for diseases in the past. Hippocrates, who is considered the father of modern medicine once wrote, “To eat when you are sick, is to feed your illness”. The ancient Greeks believed that fasting improves cognitive abilities. Plutarch, an ancient Greek writer wrote, “Instead of using medicine, better fast today”. Other intellectual figures were also strong proponents of fasting. Philip Paracelsus, the founder of toxicology and one of three fathers of modern Western medicine (along with Hippocrates and Galen) wrote, “Fasting is the greatest remedy—the physician within”. Benjamin Franklin (1706–1790), a founding father of the United States wrote of fasting, “The best of all medicines is resting and fasting”. Finally, fasting is widely practiced for spiritual purposes and remains a part of virtually every major religion [14].

While entering into the discussion about intermittent fasting, it is important to understand the difference between caloric restriction and intermittent fasting. Calorie restriction (CR) is a reduction in total caloric intake that does not result in malnutrition. This has consistently been found to result in decreased body weight and increased longevity in many species, including non-human primates [15]. Among overweight humans, short-term CR (6-months) has been shown to significantly improve multiple cardiovascular risk factors, insulin sensitivity, and mitochondrial function. Likely due to these physiological changes, clinical trials indicate CR may have a number of beneficial effects among overweight adults, in addition to weight loss. However, over the past several decades, obesity intervention trials have revealed that the vast majority of individuals experience difficulties sustaining daily CR for extended periods of time [15].

Intermittent fasting, on the other hand, refers to eating patterns that target a pattern of dedicated periods of time (ranging from 12 h to several days) with consumption of little or no calories [3]. It is voluntary, has a fixed duration, and caloric drinks are consumed regularly. It has been found to more likely mimic human eating patterns. Intermittent fasting has had increasing popularity as an alternative to continuous CR, with emerging data supporting promise in delivering similar benefits with respect to weight loss and cardiometabolic health. As opposed to traditional CR paradigms, IF is a only requires fasting for discrete periods of time [15].

## 5. Approaches to Intermittent Fasting

There have been several proposed protocols/methods of intermittent fasting that are in practice nowadays. Some of the common ones include alternate day fasting that involves fasting for 24 h every other day, and the 5:2 method that involves a fast of 24 h twice a week and a very low-calorie diet consumed 2 other days of the week. Fasting could be on consecutive or nonconsecutive days in this method. Time restricted feed is another popular approach in which fasting occurs every day with variable hours, but usually the feeding occurs during a 6-h period with breakfast in the morning and dinner prior to 3 p.m., allowing for the duration of the fast to be 14–18 h [15,16]. These approaches and other less popular fasting approaches are shown in Table 1.

Before going into the details of the physiology of the main metabolic events that occur during the fasting period, it would be pertinent to discuss the basic research done in recent years regarding the mechanistic benefits of intermittent fasting. It has been proposed in the literature that the main mechanism that is responsible for maintaining a metabolic homeostasis is the innate circadian rhythm. During a 24-h time period, the circadian rhythm is the driving force behind coordinating a balance between the anabolic and catabolic activities. The daily fed-fast cyclical rhythm is important for maintaining the balance between mRNA and proteins, which in turn then controls multiple aspects of metabolism including glycolysis, protein synthesis, lipid synthesis and oxidation, gluconeogenesis, and mitochondrial activities [17,18]. Hence, naturally, whenever there is a disarray between circadian timing and daily food intake pattern, this can lead to a disruption in the circadian rhythm, which can then negatively impact the metabolic system. This includes increased oxidative stress, increased insulin resistance, and impaired hormonal secretion [19].

There have been a few mice model-based studies that suggested the importance of intermittent fasting in maintaining a healthy circadian rhythm, which then regulates metabolic processes. Intermittent fasting via a pattern of time restricted eating allows for a consistent period of fasting daily that alleviates the circadian rhythm disruption and subsequently improves metabolic balance. Time-restricted eating has been shown to have favorable effects on organ systems of mice (white adipose tissue, brown adipose tissue, and gut) as well as insects (brain, heart, and muscle). Moreover, in animal models, time-restricted eating also prevented glucose intolerance, fatty liver, and dyslipidemia [20,21,22]. Another study by Chaix et al. showed beneficial aspects of time restricted feeding on the metabolism of mice [23].

Several small-sized human studies have also been conducted in the recent years showing the importance of a time-restricted eating pattern towards maintaining a healthy metabolism. Most of these studies reiterated the similar beneficial aspects of intermittent fasting on various aspects of human metabolism as were demonstrated in the animal studies. Time-restricted eating resulted in a decreased energy intake, body weight, body fat, BP, blood glucose, TG, glucose tolerance, and inflammatory markers [24,25,26,27,28].

## 6. Physiology of Intermittent Fasting

When talking about intermittent fasting and its effect on health, it is important to understand the basic physiology of glucose and lipid metabolism and the concept of “metabolic switching” that occurs during the fasting state.

Randle and colleagues in 1963 proposed a theory of energy metabolism during feeding and fasting, known as the “glucose–fatty acid cycle”, in which glucose and fatty acids compete for oxidation [29]. The fed-fast cycle has four stages, namely the fed state, the post absorptive state/early fasting state, the fasting state, and the starvation or long-term fast state [30]. Glucose is the primary energy source for most tissues during the day. After meals, glucose is utilized for energy and fat is stored as triglycerides in adipose tissue. During prolonged periods of fasting, triglycerides from adipose tissue are converted to fatty acids and glycerol, which are subsequently metabolized for energy. The liver then converts fatty acids to ketone bodies, which during fasting become a major source of energy for many tissues especially brain. The fed and post-absorptive states are the only times relevant to normal eating routines. In the intermittent fasting regimen, an individual often goes through the fed, post-absorptive, and fasting states. Insulin is the main driver hormone in the feeding state, where the body uses glucose as a fuel, whereas in the fasting state glucagon is the primary hormone and the body uses liver stores of glycogen for energy. The onset of metabolic switch is the point of negative energy balance at which liver glycogen stores are depleted and fatty acids are metabolized. This typically occurs beyond 12 h after the cessation of food intake. The metabolic switch from utilizing glucose to fatty acid-derived ketones represents an evolutionarily trigger shifting metabolism from lipid/cholesterol synthesis and fat storage to the mobilization of fat through fatty acid oxidation and fatty-acid derived ketones, preserving both muscle mass and function. Thus, it has been hypothesized that intermittent fasting regimens that induce this metabolic switch have potential to improve body composition in overweight individuals [16].

**Figure 1:**
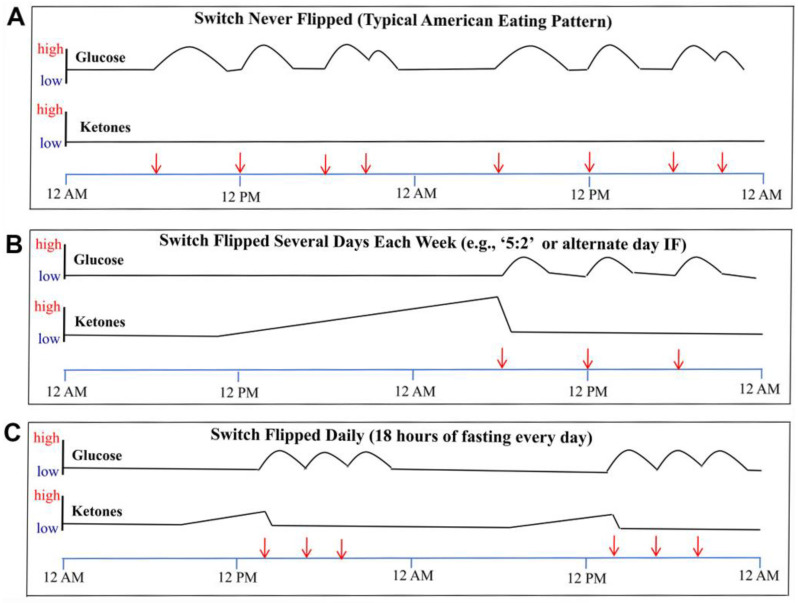
Glucose and ketone levels over the course of three meal eating patterns. (**A**) The standard meal pattern of three meals daily does not result in an appreciable rise in ketone levels. (**B**) The 5:2 or alternate-day fasting pattern allows ketones to rise during prolonged fasting, followed by suppressed ketones during the typical feeding day. (**C**) When meals are compressed to a 6-h period each day, ketones are able to rise during the time between feeding periods. From Anton et al., Obesity 2018: 26(2): 254–268; used by permission [16].

To further understand the concept of metabolic switching, let us review graphical examples of the profiles of circulating glucose and ketone levels over 48 h in individuals with a typical American eating pattern or two different intermittent fasting patterns of eating [16]. Figure 1A shows that when individuals consume three meals plus snacks every day, the metabolic switch is never initiated and their ketones remain low and the total utilization of glucose is much higher than among individuals on an intermittent fasting eating pattern. In Figure 1B, we see an example of a person who fasted completely on one day then had three separate meals on the subsequent day. On the day involving fasting, ketones are progressively elevated and glucose levels are consistently low, while on the day with food consumption, ketones remain low and glucose levels are elevated around meal consumption. Finally, Figure 1C highlights an example of a person who consumes all of their food within a 6-h time window each day. In this case, the metabolic switch is flipped during the following 12 h of fasting and remains on for approximately six hours each day, until food is consumed after approximately 18 h of fasting [16].

Metabolic switching through intermittent fasting results in improved metabolism, increased health span, and increased longevity through multiple processes [16,30]. Pathways mediating these effects include rising AMP (and ADP) and decreasing cellular ATP, resulting in the activation of AMP-activated protein kinase (AMPK)—ultimately inhibiting multiple anabolic pathways and stimulating catabolic reactions autophagy, thereby eliminating damaged proteins and organelles, and improving mitochondrial function. A decrease in circulating amino acids and glucose inhibits mTOR and leads to decreased protein synthesis, and an increased mitochondrial biogenesis and autophagy—resulting in prolonged life span in experimental animals [16]. Through reduced carbohydrate intake, fasting depletes liver glycogen, mobilizes fatty acids from adipose tissues, and stimulates hepatic β-oxidation with a rise in ketone production (β-hydroxybutyrate). Additionally, the NAD+ deacetylase activity of sirtuins is activated, resulting in autophagy and reduced oxidative stress. Together, these pathways lead to longevity and improved health span [16,30].

Free fatty acids also activate the transcription factors peroxisome proliferator–activated receptor α (PPAR-α) and activating transcription factor 4 (ATF4), resulting in the manufacture and circulation of fibroblast growth factor 21 (FGF21), a protein with far-reaching effects on various cell types throughout the body and brain [16,30]. β-hydroxybuterate also has signaling functions, including the activation of transcription factors including cyclic AMP response element–binding protein (CREB) and nuclear factor κB (NF-κB) and the expression of brain-derived neurotrophic factor (BDNF) in neurons [16,30].

In clinical studies, intermittent fasting regimens have been mainly targeted counteracting obesity and maximizing effects on healthy living by reducing progression to cardiovascular disease, MetS, hypertension, and T2DM. Importantly, there have not been a sufficient number of long-term comparative studies to indicate whether one of these dietary protocols results in long-term efficacy. Here, we will review the current literature on various effects of intermittent fasting on weight loss, insulin resistance, cardiovascular parameters, and oxidative stress/inflammation.

## 7. Intermittent Fasting and Weight Loss

Visceral adipose tissue functions as both a paracrine and endocrine organ via adipokine secretion. These adipokines are either proinflammatory, (e.g., leptin), or anti-inflammatory (e.g., adiponectin) [31]. Leptin plays a role in regulating of body weight through altered signaling to the hypothalamus and other brain regions, suppressing food intake and increasing energy expenditure. Adiponectin acts on various receptors, resulting in increased skeletal muscle and hepatic fatty acid oxidation, reduced hepatic gluconeogenesis, and increased glucose uptake. Adiponectin levels decrease in proportion to the accumulation of visceral fat. Beneficial effects of intermittent fasting in terms of obesity are at least partly due to the shift during fasting from the utilization of glucose to fatty acids and ketones as the preferred fuel source. Intermittent fasting has been shown to reduce adiposity; this is particularly from visceral fat and truncal fat because of relatively minor energy deficits. Because of this reduction in adiposity, patients may experience improvements in their leptin/adiponectin levels and sensitivity, resulting in to improved appetite control [32].

Trepanowski et al., 2017 [33], compared the effects of alternate-day fasting vs. daily calorie restriction on weight loss, weight maintenance, and risk indicators for cardiovascular disease. This was a randomized clinical trial of obese adults (18 to 64 years of age; mean body mass index, 34) at a single-center academic institution in Chicago. Participating individuals were randomized to one of three groups for one year: alternate-day fasting (25% of energy needs on fast days; 125% of energy needs on alternating “feast days”), calorie restriction (75% of energy needs every day), or a no-intervention control. The primary outcome was a change in body weight. Secondary outcomes were adherence to the dietary intervention and risk indicators for cardiovascular disease. Over the course of 12 months, the control group maintained their weight around the baseline weight, while the alternate-day fasting and daily caloric restriction groups, by 6 months, both exhibited a nadir in weight 7% below the baseline weight (not significantly different from each other before exhibiting some later weight gain, ending at 12 months approximately 4.5% below starting weight [33]. There was a 38% dropout in the alternate-day fasting group compared to 29% in the daily calorie restriction group and 26% in the control group. Overall, the authors concluded that alternate-day fasting did not improve health beyond calorie restriction [33].

## 8. Intermittent Fasting and Insulin Resistance, Diabetes and Prediabetes

There are several proposed mechanisms regarding the development of insulin resistance. One prominent theory relates to associations between increased adiposity and subsequent chronic inflammation, leading to the development of insulin resistance in tissues [34]. Intermittent fasting can decrease adiposity and related insulin resistance through reduced caloric intake and metabolic reprogramming. Another hypothesis is that decreased energy intake, such as that achieved through intermittent fasting, will cause a prolonged decrease in insulin production and increased levels of AMPK, which likely plays a role in the improvements in insulin sensitivity and glucose homeostasis [34].

**Figure 2:**
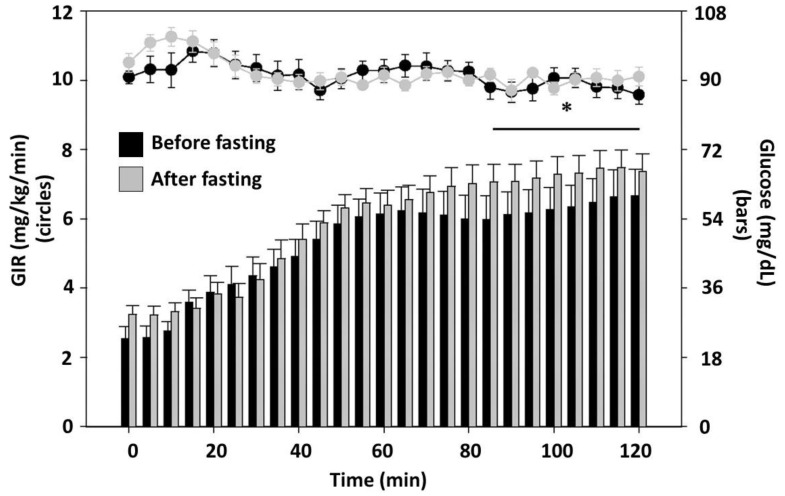
Glucose infusion rate (GIR) and glucose level during hyperinsulinemic clamp before and after 20 days of intermittent fasting. Participants were eight healthy males who had a hyperinsulinemic clamp performed before and after 20 days of alternating-day fasting for 20 h. Left axis (bars) shows the glucose infusion rate (GIR) necessary to maintain euglycemia during both clamps. Right axis (dots) shows the plasma glucose concentrations. Black bars and dots represent data from the clamp before the fasting intervention; gray bars and dots are data from after the fasting intervention. Following fasting, the insulin-mediated glucose update increased from 6.3 ± 0.6 to 7.3 ± 0.3 mg/kg/min. * *p* < 0.05 for comparing after fasting values to before fasting values. From Halberg et al. J Appl Phys 2005: 99(6):2128–2136; used by permission [35].

Halberg et al. conducted a study on the effects of fasting on insulin sensitivity [35]. The study included eight healthy young men with a mean BMI of 25.7. The subjects performed intermittent fasting for 20 h on alternate days for 14 days with a steady amount of physical activity. Interestingly, the reported weight loss was not significantly different, but fasting effected the insulin sensitivity, as illustrated in Figure 2. The glucose infusion rate and glucose concentrations during hyperinsulinemic clamp studies before and after intermittent fasting were compared. The glucose infusion rate (GIR) necessary to maintain euglycemia increased during the last 30 min of the clamp study increased after the fasting intervention compared with before, with insulin-mediated whole body glucose uptake rates increasing from 6.3 ± 0.6 to 7.3 ± 0.3 mg/min/kg, respectively (*p* = 0.03). In addition, the insulin-induced inhibition of adipose tissue lipolysis was more prominent after than before the intervention (*p* = 0.05). Following the 20-h fasting periods, adiponectin levels were increased compared with the basal levels before and after the intervention (5922 ± 991 vs. 3860 ± 784 ng/mL, *p* = 0.02). This was the first clinical research experiment to demonstrate that intermittent fasting increases rates of insulin-mediated glucose uptake.

Several clinical trials are available in the literature that have reviewed the effects of intermittent fasting on insulin sensitivity, including those assessing alternate-day fasting, [36,37,38], alternate-day modified fasting protocols, [33,35,39,40,41,42,43,44,45,46], and those using the 5:2 method of intermittent fasting [47,48,49,50]. These overall demonstrate improvements in insulin sensitivity and reductions in dyslipidemia, although not all of the studies reported all of these findings.

One interesting study that looked at the effects of intermittent fasting on insulin sensitivity was done by Sutton et al. [51]. The aim of the study was to test whether intermittent fasting can have benefits independent of weight loss. This trial used a relatively new form of intermittent fasting called time-restricted feeding. This was a five-week, randomized, crossover, isocaloric and eucaloric controlled feeding trial that tested early time restricted feeding (eTRF) in men with prediabetes. Participants adopted an eTRF schedule (6-h daily eating period, with dinner before 15:00 p.m.) and a control schedule (12-h eating period) for five weeks each. Primary endpoints were glucose tolerance, postprandial insulin, and insulin sensitivity assessed using a 3-h oral glucose tolerance test (OGTT). Secondary endpoints were cardiovascular risk factors and markers of inflammation and oxidative stress. Participants had a 3-h OGTT with baseline measurments in the morning and post-intervention for each study arm. Five weeks of eTRF did not affect the fasting glucose (*p* = 0.49) or glucose levels at any time point during the 3-h OGTT (*p* ≥ 0.13). However, eTRF did affect insulin levels. eTRF decreased the fasting insulin (*p* = 0.05) and decreased insulin levels at t = 60 min and 90 min post-load (*p* ≤ 0.01). Overall, eTRF reduced average and peak insulin levels by 26 ± 9 mU/L (*p* = 0.01) and 35 ± 13 mU/L (*p* = 0.01), respectively. This study also investigated the impact of eTRF on OGTT-derived indices of β cell responsiveness and insulin resistance. eTRF increased the insulinogenic index, a marker of β cell responsiveness, (*p* = 0.05), and decreased insulin resistance, as measured by the 3-h incremental AUC ratio, by *p* = 0.005. Thus, although five weeks of eTRF did not improve glucose levels, it dramatically lowered insulin levels and improved insulin sensitivity and β cell responsiveness [52]. This is consistent with multiple other clinical trials, suggesting that suggest that intermittent fasting may be more effective at reducing plasma insulin and increasing insulin sensitivity than at lowering glucose levels.

Carter et al. conducted one of the largest trials to assess the effect of intermittent fasting compared with continuous energy restricted diet on glycemic control in patients with T2DM [53]. This study was conducted over 52 weeks and included 137 individuals with obesity and T2D, at an average age of 61 years and mean baseline HbA1c of 7.3%. Subjects were randomized to either the 5:2 IER group (500–600 kcal/day for 2 days per week and normal diet every other day) or the CER group (1200–1500 kcal/day). There was a completion rate of 70%, which was similar in both groups. After 12 months of intervention, both groups displayed similar reductions in HbA1C levels (by 0.3%) and greater reductions in weight in the intermittent energy restriction group (by 6.8 kg).

A few case series have also been reported that looked at the effects of alternate-day fasting on T2DM. Some of them are shown in Table 2.

## 9. Intermittent Fasting and Cardiovascular Risk

Insulin resistance is associated with a state of increased inflammation, including elevated C-reactive protein, decreased adiponectin, lower low-density lipoprotein (LDL) particle size, and additional metabolic factors that ultimately contribute to or are associated with development of atherosclerosis and coronary artery disease [54]. Furthermore, insulin is both associated with atherogenic dyslipidemia and also increases the risk of fluid retention and congestive heart failure [55]. Thus, reducing insulin levels through intermittent fasting would be expected to reduce major adverse cardiovascular events.

### 9.1. Lipids—Mechanism of Action

The mechanisms of action by which intermittent fasting may improve the blood lipid profile were recently outlined [56]. Prior research has demonstrated nuclear expression of peroxisome proliferator-activated receptor-α and peroxisome proliferator-activated receptor γ coactivator 1 α in the liver lead to increased fatty acid oxidation and Apo A production, while simultaneously decreasing ApoB synthesis. Additionally, there is elevated fatty acid oxidation and decreased hepatic triglycerides and very low-density lipoprotein (VLDL) production. Altogether, these physiologic changes may help reduce serum levels of VLDL, LDL-C, and small dense LDL-C (sdLDL) [56].

### 9.2. HTN—Mechanism of Action

The mechanism of reduced blood pressure in the setting of intermittent fasting may be associated with the activation of the parasympathetic system, driven by an increase in the activity of the cholinergic neurons of the brainstem [3]. While brain-derived neurotrophic factor (BDNF) is primarily produced in response to glutamatergic receptor activation, studies have demonstrated that intermittent fasting is a key environmental stimulus [57].

### 9.3. Inflammation—Mechanism of Action

The concentration of adiponectin decreases during multiple pathological processes, including atherosclerosis, insulin resistance, T2DM, and coronary disease. In response to intermittent fasting, there is an increase in adiponectin secretion from adipocytes [3]. Adiponectin exhibits both anti-atherosclerotic and anti-inflammatory effects via inhibition of adhesion of monocytes to endothelial cells. Adiponectin also inhibits the release of vascular cell adhesion molecule 1 (VCAM-1), endothelial-leukocyte adhesion molecule 1 (ELAM-1), and intracellular adhesive molecule 1 (ICAM-1) on vascular endothelial cells, with an overall result of reducing local and systemic inflammation [3].

Table 3 summarizes the effects of intermittent on cardiovascular parameters [3,32]. While many of these studies give promising results on the effects of intermittent fasting, it is important to acknowledge the gaps in literature. Some noteworthy limitations of these studies include small sample sized studies with not enough randomized study designs, meta-analyses, or systemic reviews available currently. Many of the RCT’s fail to include patients with type I diabetes mellitus, who may also benefit. This is an area where further research is needed, especially incorporating the use of diabetes technology to limit the adverse events. Long-term follow up studies are also needed to assess if intermittent fasting is a sustainable lifestyle choice or not over a period of years. Patient adherence remains a problem, so this needs to be considered when devising research protocols.

## 10. Feasibility of Intermittent Fasting in Modern Lifestyle

Although this review gives a detailed narrative on the benefits of intermittent fasting on metabolic health, whether such dietary adaptation is feasible in day-to-day life remains a valid question. Fasting can be challenging and hence its practicality in the modern lifestyle can sometimes be debatable. An argument in favor of intermittent fasting can be that as there are various protocols for intermittent fasting, it is more adaptable in comparison to other restrictive dietary behaviors such as ketogenic diet, vegan diet, or daily caloric restriction. Furthermore, as intermittent fasting correlates with the natural circadian rhythm, it may be a more physiological diet. Tinsley et al. conducted a study to assess the applicability of time-restricted eating on the development of lifestyle dependent diseases in a working population. The results of the study showed that not only is this eating pattern feasible, but it also improves quality of life [60]. Another study by Wegman et al. explored the practicality of intermittent fasting in humans [61]. Participants were found to be adherent to the intermittent fasting dietary pattern, as indicated by the study surveys [2]. In general, intermittent fasting strategies were found to be relatively practical in daily life in terms of improving metabolic health. However, due to the paucity of studies on the feasibility of intermittent fasting in the modern lifestyle, further research is needed that should ideally focus on intermittent fasting and the factors related to an individual’s social influences, environment, and unique physiology.

## 11. Intermittent Fasting—Reason for Caution

Despite the promising outcomes of intermittent fasting, it does not come without side effects. Unfortunately, evidence documenting the ill effects of intermittent fasting regimens is sparse, primarily because the duration of assessing intermittent fasting regimens is weeks to months. Some of the commonly reported adverse effects include hypoglycemia, dizziness, and weakness.

Overall, hypoglycemia appears to be the most onerous side effect of intermittent fasting. Beshyah et al. conducted a cross-sectional multi-country observational study to describe the risk of hypoglycemia during periods of intermittent fasting observed in the month of Ramadan [62]. The study showed that intermittent fasting via reduced caloric intake may lead to severe hypoglycemia. This effect was further aggravated with the simultaneous use of antidiabetic drugs [62]. A review by Dardano et al. described that the fluctuating glucose concentration in the elderly was associated with increased instability in ambulation, leading to frequent falls and fractures [63]. The ACCORD trial a higher incidence of adverse cardiovascular events during episodes of hypoglycemia [64].

In addition, fasting without proper protein replacement is a well-known cause of muscle wasting and should be avoided [32]. There is potential for fasting to be dangerous, and, as such, it is not recommended for individuals with hormonal imbalances, pregnant and breastfeeding women, young children, adults of advanced age, and individuals with immune deficiencies, including those with a history of solid organ transplant with subsequent medical immunosuppression [3]. Individuals with eating disorders or those with dementia have other unique challenges that will likely be exacerbated by pursuing planned fasting and therefore should avoid intermittent fasting regimens.

## 12. Conclusions

In summary, intermittent fasting has shown positive effects on weight loss, in addition to reducing insulin resistance and favorably shifting the levels of leptin and adiponectin. Pre-clinical and clinical studies have demonstrated that intermittent fasting has a wide range of benefits for many diseases, including obesity, T2DM, and hypertension, and in improving cardiovascular risk factors. One argument against intermittent fasting is that, despite extensive animal data, many clinical trials have failed to show as significant improvements of intermittent fasting over caloric restriction. In the absence of large randomized controlled trials testing the efficacy and side effects of intermittent fasting in individuals with MetS, prediabetes and T2DM for extended durations, it is difficult to ascertain the risk to benefit ratio of the different intermittent fasting regimens. Studies assessing long-term follow-up are needed for assessing efficacy as a permanent life style approach.

## Figures and Tables

**Table 1:** Examples of approaches to intermittent fasting.

Protocol	Frequency	Duration	Additional Considerations
Alternate day	Every other day	24 h	
5:2	Two days weekly	24 h each day	2 other days involve a very low calorie diet
Time-restricted feeding	Every day	14–18 h	Food is consumed over a 6-h period
B2 regimen	Everyday	14 h	2 large meals per day: breakfast between 06:00 a.m. and 10:00 a.m. and lunch between 12:00 p.m. and 04:00 p.m. and no dinner
Weekly 1 day Fasting	Once a week	24 h	Water-only diet 1 day per week and regular eating on the other 6 days of the week
Intermittent VLCD therapy	Variable	24 h	1-d VLCD: VLCD for 1 day a week and 5-d VLCD: VLCD for 5 consecutive days a week, repeated every 5 weeks. *

* Please note that although commonly-used, intermittent very low-calorie diet (VLCD) therapy is not an intermittent fasting approach as the subject consumes a very low caloric dietary intake daily without any fasting period. This was a misnormer commonly associated with intermittent fasting.

**Table 2:** Changes in body weight and HbA1c following alternate-day intermittent fasting.

Case Series	Effects of IF vs. Baseline
Furmli et al. (7 months)	Baseline Values: HbA1C: 11%, 96.7 mmol/mol Body weight: 83.8 kg After 7 months of intermittent fasting: - HbA1C decreased by 4% - Body weight decreased by 10 kg
Furmli et al. (11 months)	Average Baseline Values: HbA1C: 6.8%, 50.8 mmol/mol Body weight: 97.1 kg After 11 months of intermittent fasting: - HbA1C decreased by 1.2% - Body weight decreased by 10.6 kg
Lichtash et al. (14 months)	Average Baseline Values HbA1C: 9.3% Body Weight: 55.3 kg After 14 months of intermittent fasting: - HbA1C decreased by 3.5% - Body weight decreased by 1.8 kg

**Table 3:** Summary of studies employing intermittent fasting, with outcome data regarding lipid, blood pressure, and inflammatory markers.

Authors (Year)	Number Enrolled	Study Design/Fasting Protocol Used	Description of Participants	Study Duration	Effect on Lipids	Effect on BP	Effect on Inflammatory Markers
Harvie et al. (2011) [48]	107	RCT Intermittent energy restriction	Overweight or obese women (premenopausal)	6 months	↓TC (*p* < 0.01); NS: LDL, TGs, HDL	↓Systolic (*p* = 0.99); ↓Diastolic (*p* = 0.84)	
Varady et al. (2013) [40]	15	RCT Alternate day fasting	Individuals with BMI 20–29.9 kg/m^2^	12 weeks	↓LDL (*p* < 0.01); ↓TGs (*p* < 0.01); NS: HDL	↓ (*p* = 0.51)	↓CRP (*p* = 0.01) ↓Leptin (*p* = 0.03) ↑Adiponectin (*p* < 0.01)
Bhutani et al. (2013) [39]	83	RCT Alternate day fasting plus endurance exercise (exercise control)	Individuals with obesity BMI 30–39.9 kg/m^2^	12 weeks	↓LDL (*p* < 0.05); ↑HDL (*p* < 0.05); NS: TC, TGs	↓Systolic (*p* = 0.254); ↓Diastolic (*p* = 0.570)	NS CRP
Eshghinia et al. (2013) [41]	15	Observation over 8 weeks with alternating day fasting	Overweight or obese women BMI ≥ 25 kg/m^2^	8 weeks	NS: LDL, TGs, HDL	↓Systolic (*p* < 0.001)	
Teng et al. (2013) [58]	28	RCT Fasting caloric restriction (300–500 cal/day) vs. control	Men in Malaysia BMI 23–29.9 kg/m^2^	12 weeks	↓TC (*p* < 0.001) ↓LDL (*p* < 0.05) NS: HDL, TGs	↓Systolic (*p* < 0.05); ↓Diastolic (*p* < 0.05)	
Harvie et al. (2013) [49]	77	RCT Intermittent energy and carbohydrate restriction vs. control	Overweight or obese women	3 months	NS: LDL, TGs, HDL		NS: IL6, TNFα, leptin, adiponectin
Erdem et al. (2018) [59]	60	Prospective cohort (observational study)	Individuals from the Cappadocia cohort with prehypertension and hypertension (SBP 120–139 and ≥140; DBP 80–89 and ≥90 mmHg	At least 1 week		↓Systolic (*p* < 0.001); ↓Diastolic (*p* < 0.039)	
Hoddy et al. (2016) [43]	59	8 week alternating day fasting Protocol	Obese individuals BMI 30–39.9 kg/m^2^	8 weeks			↓Leptin (*p* < 0.05)

BP, blood pressure; CRP, C-reactive protein; NS: not statistically significant; RCT, randomized controlled trial; TG, triglycerides; TNF-α, tumor necrosis factor-alph. ↑, increase. ↓, decreases.

## Data Availability

Please see individual studies referenced for data availability.
